# Supplementary material for: The Construction and Exploration of a Comprehensive MicroRNA Centered Regulatory Network in Foxtail Millet (Setaria italica L.)
Source: Front Plant Sci. 2022 May 6;13:848474. doi: 10.3389/fpls.2022.848474 (PMC9121102; doi:10.3389/fpls.2022.848474)
Supplement: Supplementary file 2 [file Data_Sheet_2.zip › Sit-miR172a_Seita.3G311900.1_6873_TPlot.pdf]

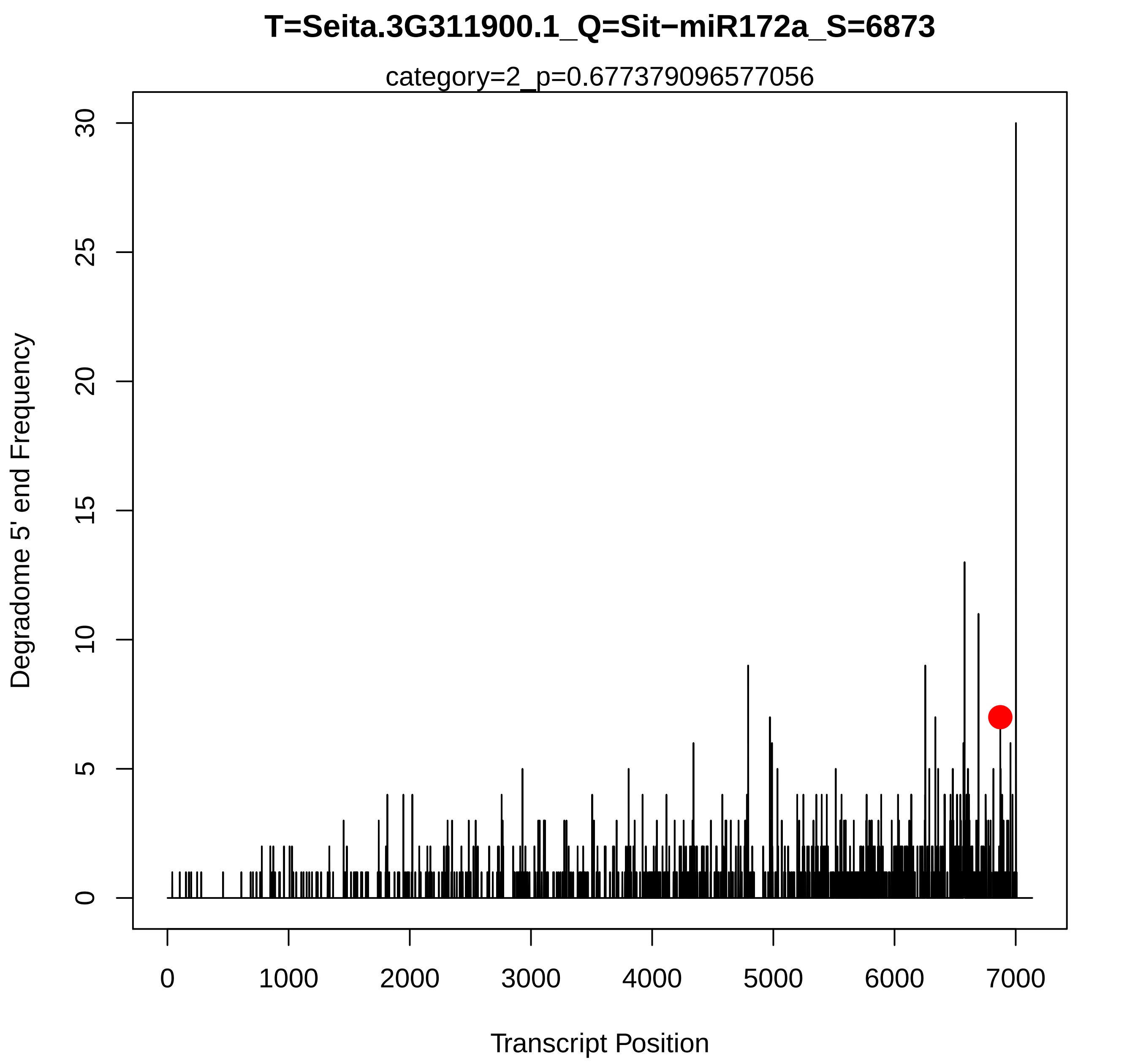

T=Seita.3G311900.1_Q=Sit−miR172a_S=6873
category=2_p=0.677379096577056
Degradome 5' end Frequency
0
5
10
15
20
25
30
0
1000
2000
3000
4000
5000
6000
7000
Transcript Position
